# Insects as Feed for Farmed Poultry: Are Italian Consumers Ready to Embrace This Innovation?

**DOI:** 10.3390/insects12050435

**Published:** 2021-05-12

**Authors:** Davide Menozzi, Giovanni Sogari, Cristina Mora, Marta Gariglio, Laura Gasco, Achille Schiavone

**Affiliations:** 1 Department of Food and Drug, University of Parma, Parco Area delle Scienze 45/A, 43124 Parma, Italy; davide.menozzi@unipr.it (D.M.); cristina.mora@unipr.it (C.M.); 2 Department of Veterinary Science, University of Turin, Largo Paolo Braccini 2, 10095 Grugliasco, Italy; marta.gariglio@unito.it (M.G.); achille.schiavone@unito.it (A.S.); 3 Department of Agricultural, Forest, and Food Sciences, University of Turin, Largo Paolo Braccini 2, 10095 Grugliasco, Italy; laura.gasco@unito.it

**Keywords:** animal welfare, attitude, consumer behavior, duck meat, insect meal, insect-based feed, intention, information, preferences, sustainability, willingness to pay

## Abstract

**Simple Summary:**

Research into alternative protein sources might help to reduce environmental pollution and increase animal welfare. Insect proteins used in feed production could represent a good solution for these environmental and ethical problems. However, consumer acceptance of insects as feed must be carefully considered, along with the role of information in affecting the acceptance of such feed. In this study, we tested how non-technical information on the benefits of introducing insects as feed for farmed animals convinced a group of Italian consumers to accept duck meat fed either with insect-based meal or live insects. We found that providing information on the environmental, safety, nutritional, and taste-related aspects of insect-based feed as a protein substitute in the poultry sector increased the consumers’ acceptance of using insects as feed, as well as their readiness to purchase and consume these products. Our results show that some sociodemographic attributes, i.e., gender, age, and education level, are significantly related with the acceptance of products from insect-fed animals.

**Abstract:**

The inclusion of insects as a protein source in feed production is not only related to technical, economical, and normative restrictions but is also affected by consumer acceptance. In this study, we evaluated consumers’ attitudes, intention to purchase and eat, and willingness to pay for meat obtained from a farmed duck fed with insect-based meal or a live insect diet. We conducted a survey among a sample of 565 consumers to test the effects of information about the benefits of using insects as feed on consumers’ attitudes towards animal-based products fed with insects. Providing information on the sustainability and nutritional benefits of using insects as feed increased both attitude towards and intention to purchase and eat meat products made from animals fed with insects. In the treatment group, we found a significant reduction from 21.9 to 14.0% in those who wanted to be compensated for buying a duck fed with an insect-based meal and an increase in those willing to pay the same price—from 64.9 to 72.7%. The information treatment significantly increased the intention to eat such products, suggesting that increasing consumers’ knowledge might help in reducing the fears and misconceptions around the topic of using insects as a feed source.

## 1. Introduction

In the context of the growing world population [1] alongside the increase in attention on animal welfare among consumers [2,3], research on alternative protein sources that help to reduce environmental pollution and increase animal welfare is becoming increasingly necessary. Insect proteins could represent a good solution for these environmental and ethical problems. Indeed, some insect species (e.g., Diptera) are able to biodegrade bio-waste or manure and provide very high feed conversion efficiency, resulting in fast lifecycles [4,5]. Moreover, insects represent a good protein source for monogastric animals (such as poultry) and are also part of the natural diets of many wild animals, including birds [6,7]. On the other hand, insect larvae are rich in fats, which can be partially or totally removed from the insect meal. These lipids, which are not included in the feed ban regulation [8], can be applied in animal nutrition to provide energy and valuable fatty acids. Insects can be easily included in the circular economy, giving them new application possibilities and increasing their value. Moreover, it has recently been shown that insects contain bioactive compounds that are charged to have positive effects on animal health and welfare [9,10]. According to EU regulations, insects kept for food, feed, or other purposes are considered “farmed animals” [11] and must follow all the necessary animal regulations. Therefore, in the EU, insects can only be fed with products of a non-animal origin or products of an animal origin comprising Category 3 materials included in the catalogue of feed materials [12]. However, in the European Union, insect meals are only authorized for aquaculture feed and for pet nutrition [12], while live insects are permitted under national legislation in certain EU Member States for fish, poultry, and pig [13]. The primary reason for these restrictions is the possible occurrence of Transmissible Spongiform Encephalopathy. 

The inclusion of insects in the poultry sector is not only related to technical, economical, and normative restrictions but also to consumer responses—i.e., attitude, intention to purchase and consume, and willingness to pay for insect-fed animals. Therefore, consumer acceptance of insects as feed is crucial to determine the development of the sector of insect farming and of the foods obtained from animals fed on an insect-based diet [14].

Despite the growing literature on consumer acceptance of edible insects as foods in Western countries [15,16], few studies have examined consumers’ preferences and attitudes towards the use of insects as feed [13].

To date, most studies on consumer acceptance of insects as feed have focused on aquaculture, including fish in general [17], along with specific species of fish, such as farmed rainbow trout [18,19], Scottish salmon [20], European sea bass, gilthead sea bream, and rainbow trout [21]. Few studies have evaluated respondents’ opinions on accepting the meat of poultry fed with insects. Domingues et al. [22], in a study on a Brazilian sample, showed that the factors influencing consumers’ willingness to accept the use of insects in animal feed depend on the animal species in question (e.g., poultry, pigs, cattle, or fish). The authors’ results indicated that the use of insects to feed fish was accepted more widely than the use of insect-based feed in the poultry sector. One study showed that environmentally conscious consumers preferred chicken breast produced with insect meal over other types of feed [23]. Another work suggested that consumers more readily accept the use of insects as feed (e.g., a burger made from an insect-fed chicken) than insect-based food products [24]. Spartano and Grasso [25,26] evaluated consumers’ willingness to pay for eggs from insect-fed hens in the UK market. The results suggested that most of the respondents would be willing to pay a premium for these eggs and that providing information on the potential benefits would positively influence the intention to consume and purchase.

Many past studies showed that providing information about the practice of producing and consuming insects, e.g., via educational sessions, could reduce the barriers to tasting insect-based products [27,28]. However, to the best of our knowledge, only a few studies have been published on the role of information in influencing the attitude and willingness to eat an animal fed with insects [19,20,25,29].

Therefore, this research aimed to test how the introduction of non-technical information on the benefits of using insects as feed for farmed animals would influence a group of Italian consumers in accepting duck meat fed either with insect-based meal or live insects. As suggested by previous research [19,23], we hypothesized that information on using insects as feed would translate into higher quality perception regarding sensory aspects and production sustainability, with the overall effect of overcoming negative emotions such as disgust, as well as increasing attitude, intent to purchase and consume, and willingness to pay for animals fed with insects. We also hypothesized that this acceptability would be strongly correlated with some individual characteristics such as gender and education.

## 2. Materials and Methods

### 2.1. Data Collection and Analysis

The presented study was conducted in Italy, and the data were collected between December 2019 and January 2020. Subjects were excluded with screening questions when they were (1) younger than 18 years old and/or (2) vegetarian/vegan. From a total sample of 583 participants, 18 subjects who took a short time to complete the survey (i.e., respondents who were below 50% of the median duration) were excluded to ensure high-quality data. The final sample included in the further analyses consisted of 565 respondents (53.10% female). The age range was 18–80 years, with a mean age of 38.84 years (SD = 13.86), and most of the participants indicated a “tertiary education level” (80.17%), which corresponded to a college degree or higher. The rest had a “secondary education level” (18.76%). No significant differences in demographics were found between the two groups (control and information treatment).

Furthermore, at the beginning of the questionnaire, all participants signed a consent form electronically that outlined the confidentiality and de-identification of collected data according to EU regulations. Upon review of the human subject protocol, this study was approved by the Research Ethics Committee of the University of Turin (Protocol ID: 122601).

We performed descriptive and inferential statistical analyses on the dataset and tested the normality of the data distribution with a Shapiro–Wilk test. The results were expressed as the median and interquartile range (IQR) with the mean and standard deviation (SD). Since non-normality of the data was found, we used a non-parametric Mann–Whitney test for independent samples to explore the differences in the variables between the control and treatment groups. A non-parametric Wilcoxon signed-rank test was used to compare repeated measurements in both the control and treatment groups (e.g., differences in respondents’ attitudes before and after the information treatment). Pearson’s chi-squared test and Cramer’s V were applied to explore the potential associations between nominal variables, such as the association between the willingness to pay a lower/equal/higher price for a duck leg fed with an insect-based meal in the control and treatment groups. Finally, a Spearman’s rank correlation coefficient, or Spearman’s ρ, was used to explore the correlation between age and the variables. All statistical analyses were performed using the SPSS software (Version 26.0, IBM Corporation, Armonk, NY, USA).

### 2.2. Survey Design and Questionnaire

The questionnaire was administrated and web-programmed in Qualtrics^®^ and was spread using common social media channels. The questionnaire was written in English and then translated into Italian with the collaboration of an English–Italian teacher with expertise in Food Science vocabulary. The questionnaire was structured with closed-ended questions and divided into six parts. 

In the Section 1, respondents were presented with four statements concerning their willingness to purchase (from 1—extremely unlikely to 7—extremely likely) a duck fed with either cereals, genetically modified (GM) soybean, non-GM soybean, fish meal, or insect meal. 

The second part of the survey asked the respondents about their attitude, purchase intention, and willingness to pay for a farmed duck fed with (1) insect meal and (2) live insects. Each respondent’s attitude pre-information treatment was measured with the following four items on a 7-point scale: “I believe that using insects as feed for ducks: negatively–positively affects the taste of the meat”, “[…] negatively–positively affects the nutrition properties of the meat”, “[…] negatively–positively affects the taste of the final duck-based products (e.g., duck sausages)”, and “[…] is extremely disgusting–not at all disgusting”. The values of these items are reported in Table A1 in Appendix A. The purchase intention was also asked before the information treatment using a single item: “What is your willing to purchase a duck fed with an insect diet?” (…). Finally, respondents were asked about their attitudes towards labeling the use of insect meal as feed [20] with a single question on a 7-point scale (from 1—strongly disagree to 7—strongly agree): “If the duck meat or duck-based products (e.g., sausages or foie gras) that I usually buy were fed with insect-meal, I would like this information to be provided on the label”.

Then, we randomly split our sample into two groups: the control group and the treatment group. The treatment group received information about the environmental benefits of insect meal before the questions were asked. The information was provided in the form of a short text adapted by Altmann, Risius, and Anders [23]; Laureati, Proserpio, Jucker, and Savoldelli [30]; and Popoff et al. [20]: “The world population is increasing along with the demand for food. Consequently, the concern for food reserves is growing. Insects are increasingly recognized as an alternative source of protein for use as animal feed. In fact, many insect species are highly nutritious, and their production has a lower environmental impact than other feed protein sources, such as soy. Therefore, it was recently proposed that the protein part of traditional feed (composed only of vegetable ingredients) used in farmed animals (e.g., ducks) could be partially replaced with products derived from insects. In addition, insects are eaten in nature by many animals such as fish, pigs, and poultry, including chickens and ducks, and can, therefore, be considered a natural food. Furthermore, no type of sensory alteration has been identified in the final products”. 

Then, after having provided the information treatment, each consumer’s attitude towards eating a farmed duck fed with insect-based meal/live insects was measured using six statements on a semantic scale: “Bad–Good”, “Unsatisfied–Satisfied”, “Unpleasant–Pleasant”, “Dull–Exciting”, “Terrible–Delightful”, and “Negative–Positive”. Then, with four items, we measured the intention to purchase a farmed duck fed with different kinds of feed, such as a vegetable meal diet, insect-based meal, and live insects, as well as the intention to purchase a wild duck. Thereafter, the respondents answered two questions about their willingness to pay for a duck leg fed with (1) insect-based meal and (2) live insects, respectively. We showed the respondents the average retail price for duck leg (8.95 €/kg), which was determined after a market inventory conducted in several different grocery stores. Three possible answers were given: “I would pay a lower price”, “I would pay the same price”, and “I would pay a higher price”. 

The fourth part gauged emotional consumer responses by using a sentence to paraphrase the meaning of the feeling [31]. Nine emotions were introduced in the question “How does it make you feel when you think of eating a duck fed with an insect diet?” and presented in a balanced order across subjects.

The Section 5 included a question adapted from Popoff et al. [20] about the willingness to eat (“Would you eat farmed duck fed on an insect-based diet?”), with the possibility to select only one option (“Yes, because…”; “Yes, but…”; “Maybe, if…”; “No, because…”), followed by an open-ended question to better explain the choice. 

Finally, in the last section, the socio-demographic information was collected, including gender, age, and educational background.

## 3. Results

### 3.1. Willingness to Purchase Duck Fed with Different Feed Sources

Table 1 shows how willing respondents would be to purchase a duck farmed with different types of feed. Normal data distribution was rejected by the Shapiro–Wilk test (*p* < 0.001). Before providing the information treatment, we found a moderately positive intention to purchase a duck fed with insect meal. The highest score was found for cereal-based feed (median = 7.00, IQR 2.00), followed by non-GM soybean meal and insect meal (median = 6.00, IQR = 3.00). The lowest scores were given to the GM soybean and fish meal, which were the least liked feed sources. 

The respondents also expressed their attitudes towards labeling. Approximately 80% replied that they would like information to be provided on the label if the duck-based products they usually buy have been fed with insect meal (mean = 5.91, SD = 1.48).

### 3.2. Effects of the Information Treatment 

Table 2 shows that participants’ attitudes towards and intention to purchase a duck fed with insects varied between the control and treatment groups. A non-parametric Mann–Whitney test was applied to explore the differences between groups since the data were non-normally distributed (Shapiro–Wilk test, *p* < 0.001). The attitude towards eating and the intention to purchase a farmed duck fed with insect-based meal, which were measured as pre-information, did not differ significantly between the control and treatment groups (with *p* = 0.240 and *p* = 0.741, respectively). However, once information was provided, the results show that, compared to the control (no information), the group treated with the information had a significantly more favorable attitude towards ducks raised with both insect-based meal (*p* < 0.001) and live insects (*p* = 0.001) and a significantly greater willingness to purchase such products (with *p* < 0.01 and *p* < 0.05, respectively). On the other hand, the respondents’ intention to purchase wild duck or farmed duck fed on a vegetable meal diet was not significantly different between the control and treatment groups (Table 2). Overall, a higher mean score in the control group was found for the intention to purchase a farmed duck fed on a vegetable meal diet, whereas in the treatment group, the intention to purchase a farmed duck fed with live insects or a duck fed with insect-based meal received higher mean scores. 

**Figure 1:**
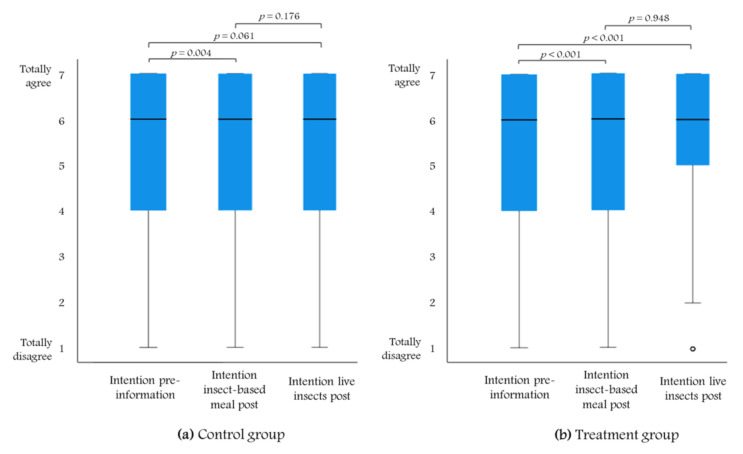
Box plot representation and Wilcoxon signed-rank test results of intention to purchase a farmed duck fed with insects pre- and post-information treatment in (**a**) the control (*n* = 279) and (**b**) the treatment groups (*n* = 286).

As shown in Figure 1, we tested the change in the intention to buy duck meat fed with insects from pre- to post-information. Although the control group did not receive any information on the use of insects, the Wilcoxon signed rank test showed that the intention was significantly higher for generic insect-based meal (*p* < 0.01) but not significantly different for live insects (*p* = 0.061). In the treatment group, however, the intention to purchase a duck fed with insect-based meal and live insects was significantly higher compared to the intention to purchase a duck fed with an insect diet before the information treatment (*p* < 0.001). This shows that information significantly affected the intention to eat a duck fed with live insects. 

**Figure 2:**
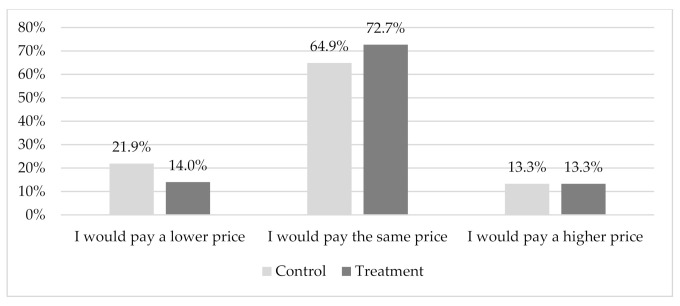
Responses to the question “Considering that the price of a duck leg fed with vegetable meal is 8.95 €/kg, how much would you be willing to pay for a duck leg fed with insect-based meal?” for the control (*n* = 279) and information-treatment groups (*n* = 286). Pearson’s chi-squared (df) = 6168 (2), *p* = 0.046; Cramer’s V = 0.104.

Finally, when considering differences in consumers’ willingness to pay (WTP) for a duck fed with an insect-based meal or live insects, no significant differences were found between the control and treatment groups. However, we observed a significant reduction (*p* = 0.046) in the respondents who wanted to be compensated for buying a duck fed with insect-based meal—i.e., those who desired to pay a lower price (from 21.9 to 14.0%) and a parallel increase in those willing to pay the same price (from 64.9 to 72.7%) (Figure 2). This reduction was not confirmed in the WTP for buying a duck fed with live insects.

### 3.3. Socio-Demographic Differences in Attitude, Intention, and Willingness to Pay

Finally, Mann–Whitney tests were performed to find socio-demographic differences in terms of attitude, intention, and WTP. The distribution between the control and treatment groups by gender, educational level, and age was homogeneous. When considering gender, males showed a more favorable attitude than females towards a farmed duck fed with live insects and a stronger intention to purchase a farmed duck fed with insect-based meal and live insects. However, as the average price for a duck leg fed with vegetable meal is 8.95 €/kg, the median WTP for a duck leg fed with insects did not significantly differ between genders (Table 3). 

When considering age, younger people had a more favorable attitude towards eating a farmed duck fed with insect-based meal and live insects, as demonstrated by the negative and significant—although weak in magnitude—correlation coefficients (respectively, Spearman’s ρ = −0.214, *p* < 0.001, and ρ = −0.152, *p* < 0.001). Similarly, younger respondents showed a slightly higher intention to purchase a farmed duck fed with insect-based meal (Spearman’s ρ = −0.086, *p* = 0.042). However, the intention to purchase a farmed duck fed with live insects and the respondents’ WTP were not significantly correlated with age. 

Finally, the Mann–Whitney tests revealed a significantly more favorable attitude towards eating a farmed duck fed with insect-based meal among respondents who completed a tertiary educational level compared to those with only secondary education (*p* = 0.047; Table 4). A higher education (tertiary vs. secondary education level) was also positively associated with the intention to purchase a farmed duck fed on an insect-based meal (*p* < 0.001) or with live insects (*p* = 0.005). The median WTP for such products was not significantly different than the base price of 8.95 €/kg, regardless of the respondent’s educational level or feeding method (i.e., insect-based meal or live insects).

### 3.4. Emotions Related to Eating a Duck Fed with an Insect Diet 

**Figure 3:**
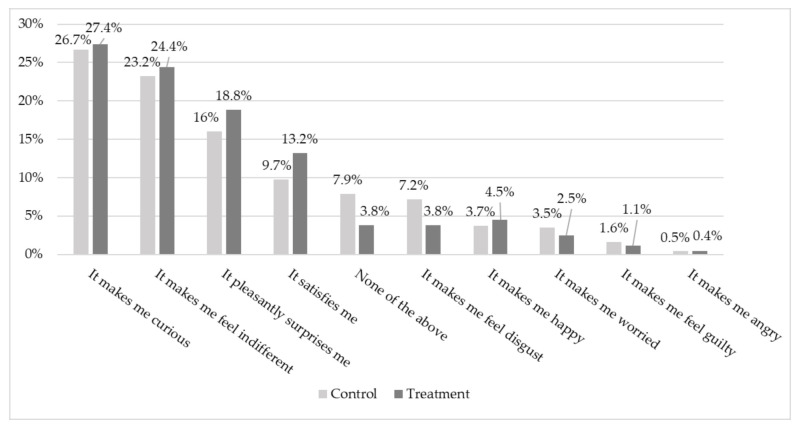
Frequency of emotional terms reported by treatment. Pearson’s chi-squared (df) = 16,625 (9), *p* = 0.065.

When asked how the idea of eating duck fed with an insect diet made them feel, most respondents reported curiosity and indifference, followed by a pleasant sense of surprise, with the informed group presenting higher percentages (27.4, 24.4, and 18.8%) than the control group (26.7, 23.2, and 16.0%), respectively, for curiosity, indifference, and pleasant sense of surprise. However, the only emotion that was significantly affected by the information was “disgust”. The frequency of “disgust” as a response decreased from 7.2% in the control group to 3.8% in the treatment group (*p* < 0.05). Figure 3 shows the frequency of emotional terms reported by treatment.

**Figure 4:**
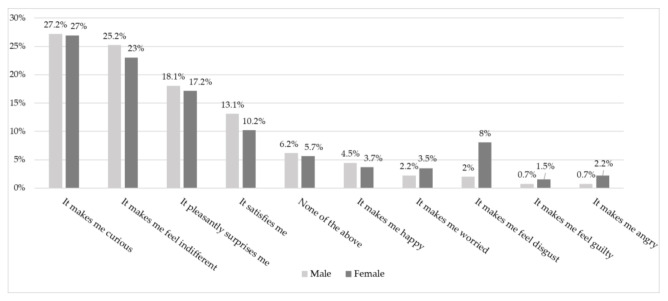
Frequency of emotional terms reported by gender. Pearson’s chi-squared (df) = 16,919 (9), *p* = 0.012.

Figure 4 illustrates how the emotional term frequency changed based on gender. No significant gender differences emerged among most of the emotions, except for disgust. As shown in the table, females associated the idea of eating a duck fed an insect diet with disgust more often than males (8.0% compared to only 2.0% for females and males, respectively (*p* < 0.001)).

### 3.5. Differences in Intention to Eat

**Figure 5:**
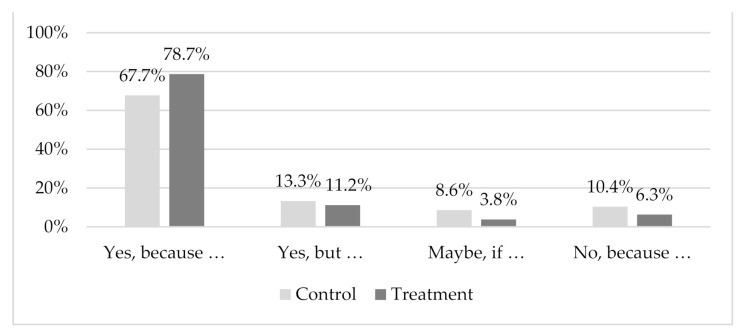
Responses to the question “Would you eat farmed duck fed an insect-based diet?” for the control (*n* = 279) and information treatment groups (*n* = 286). Note: Pearson’s chi-squared (df) = 10.811 (3), *p* = 0.013; Cramer’s V= 0.138, *p* = 0.013.

When considering potential differences in attitudes between the control and information treatment groups, we also found that respondents in the treatment group reported a significantly higher intention to eat a farmed duck fed on an insect-based diet (*p* < 0.05, Figure 5). The Pearson’s chi-squared test and Cramer’s V indicated a significant association of *p* = 0.013 between the treatment condition and the responses to the question “*Would you eat farmed duck fed an insect-based diet*?”. Most respondents were prepared to eat insect-fed duck without any concerns, especially among those provided with the information (78.3%) compared to the control group (67.7%). The respondents considered insects to be a “*natural diet*” with a “*low environmental impact*”.

The rest of the sample indicated they would be willing to eat insect-fed duck only under certain conditions (“Yes, but…” and “Maybe, if…”). In this case, participants in the treatment group also reported that eating duck fed with insect-based meal was more reasonable than those in the control group. Among the main reasons for their responses, the respondents indicated that they would consume the meat “*only if the insects are part of the life cycle of ducks*” and if “*the taste is not altered*”, further noting that “*the safety of insects should be reported*”.

Finally, only a small number of respondents were completely opposed to eating such products, mainly due to disgust (“*just thinking about that makes me disgusted*”) or a lack of knowledge (“*not sure if ducks are insectivorous*”). Most of them refused simply because they rarely consume duck meat. Table A2 in the Appendix A reports the main topics raised in the open comments.

## 4. Discussion

In the present study, we explored the main determinants related to Italian consumers’ acceptance of using insects as feed in the duck sector. More specifically, we investigated the potential role of information on the benefits of including insects as feed in influencing the emotions, intention to eat, attitude, purchase intention, and WTP for two types of insect feed for farmed duck: the use of insect-based meal and the use of live insects.

In line with the findings of Bazoche and Poret [19], our study confirms the role of information on the sustainability and nutritional benefits of using insects as feed compared to traditional feed sources in increasing both attitude towards and intention to purchase and eat meat products from animals fed with insects. While the control group reported a higher intention to purchase a duck fed with vegetable meal, respondents in the treatment group reported a higher intention to purchase a duck fed with insect feed.

Interestingly, the results show that providing information generated a larger variation (increase) in the intention to purchase duck fed with live insects than that fed with insect meal. Several factors could explain the difference observed between “insect meal” and “live insects”. First, consumers may prefer the idea that poultry animals are fed with insects because such feeding also happens in the animals’ natural environments [32]. In nature, insects are consumed alive rather than as processed insect meal. Therefore, consumers might associate eating live insects with the more natural behavior of ducks and greater animal welfare. Second, the idea that animals eat something “fresh” (i.e., alive) and not processed could be perceived as more positive for safety and nutritional reasons. Third, we must consider how we named the feed in our survey, as the term could have been perceived differently among respondents. Currently there are several ways to name processed insect feed, such as “insect meal”, “insect-based feed”, “insect-based meal”, “insect-based diet”, and others. As suggested by Bazoche and Poret [19], the term “insect meal” could decrease the acceptability of using insects as feed because this term may be too closely related to “animal meal” (e.g., “fish meal”). 

Consumer considerations regarding animal welfare were also noted in the open comments, with many respondents highlighting that they were willing to eat the insect-fed duck if the label certified how this diet increased the animal’s nutrition and improved the animal’s welfare. Today, consumers are increasingly concerned with obtaining meat and animal-based products from cattle or poultry farmed under high welfare standards [25], and the feeding system is an important animal welfare factor [33]. Indeed, when the respondents were asked about their willingness to purchase duck fed with different feed sources, the highest preference was obtained for “cereals”, which is considered a common feed source for poultry in nature, followed by non-GM soy and insect meals. Unlike the findings of Szendrő et al. [29], which showed that Hungarian consumers were not worried about animals fed with GM soy meals, our study indicated that a duck fed with GM feed was the least favorable. This could be explained by differing perceptions of GMOs in general, as previously documented [34].

In line with the findings of Popoff et al. [20] and Kulma et al. [35], most of the respondents were generally in favor of eating farmed animals fed on an insect-based diet. The information treatment significantly increased the intention to eat such products, implying that the fear and misconceptions surrounding the use of insects as a feed source are mainly due to a lack of knowledge [25]. The significant difference between the control and treatment groups suggested that the informed respondents understood and integrated the new information in their choices and preferences. Based on the open comments, awareness that insects are considered a natural type of feed for poultry can be well accepted as a driver for eating such duck meat. Another important aspect that can increase the intention of eating insect-fed animals is reassurance that the taste of the final products will not be altered. Finally, respondents liked the idea that this new type of feed offers a more sustainable alternative to tradition meal (i.e., soybeans) and can help in preserving natural resources. Only a small number of participants rejected the idea of consuming duck fed with insects due to a so-called “disgust factor”, in line with other studies on the acceptance of insects in feed [29,30] and food [36].

The importance of information emerged strongly in the open-comment questions, with most of the respondents declaring themselves to be willing to eat the insect-fed duck only if they knew more about this new method. Moreover, in line with the findings of Popoff et al. [20] on fish fed with insects, most of the respondents would appreciate the inclusion of information about the use of insect feed on the labels of meat-based products, thus ensuring greater information transparency and leading to an increase in consumer trust. 

As in other studies on the use of insects as food [27,37], the present findings confirmed that the provision of information about insects as an alternative protein source reduced the feelings of disgust surrounding the inclusion of these animals in the food supply chain. In line with the results of Bazoche and Poret [19] and Spartano and Grasso [25], we confirmed that providing information on the positive outcomes of this new feed type might help reduce the barrier to accepting insects in feed and the food chain. This finding is consistent with the theory of heuristics, which posits that the less specific information consumers have, the more the consumers will rely on their emotions [24]. Thus, a well-informed consumer will have lower feelings of disgust and greater acceptance towards the use of insects as feed. The results indicate generally positive feelings associated with including insects as feed, with curiosity and pleasant surprise as the most recurring terms reported among our sample. Therefore, curiosity could be one of the first reasons for consumers to purchase an insect-fed animal product, as also reported in studies focusing on food containing insects [38].

In contrast with the study of Ferrer Llagostera et al. [21], which found that consumers were willing to pay a premium for insect-fed fish, our results show that most of the respondents (64.9% in the control and 72.7% in the treatment group) would be willing to pay the same average price for both a duck fed with insect meal and a duck fed with vegetable meal. This might be explained by the low purchase familiarity among our respondents regarding the product under investigation (duck meat). 

Our results also show that some sociodemographic attributes (gender, age, and educational level) are significantly related to the acceptance of products from insect-fed animals [30]. In contrast with the findings of Kulma et al. [35], gender appeared to be one of these determinants, with men more willing than women to consume and purchase duck fed with both insect-based meal and live insects. This result is in line with previous study findings on insect-fed fish [18,19]. This result could be explained by the higher neophobia and disgust usually expressed by women, as found in other Italian studies focusing on insect-based foods [36,37]. Moreover, the emotion of disgust, which was linked to a fear that the final food product would taste different and other negative thoughts, was shown to be stronger among women than men. Our results also confirmed the role of education when studying consumers and insects, as also shown by Verneau et al. [39], who assessed the willingness to adopt insects as food. We found that the intention to purchase a farmed duck fed with insect-based meal and live insects was stronger among respondents with a tertiary education level [35]. In line with the findings of Kulma et al. [35], the highest willingness to eat animals fed on insects was observed among younger people.

Our main conclusion is that providing information on the environmental, safety, nutritional, and taste-related aspects of insect-based feed as a protein substitute in the poultry sector increased consumers’ acceptance of using insects as feed, as well as their readiness to purchase and consume these products. 

Past studies have shown a minimal impact on the sensory and quality characteristics of meat when the animals are farmed using a partial replacement of conventional protein sources with insect proteins [40]. Nevertheless, one of the barriers to the development of the insect industry for feed could be the low consumer motivation to accept insects as feed. However, some authors [41] suggest that using insect species as feed may be more readily accepted than using insects as food. Our results support this assumption, as disgust, which is one of the main barriers to accepting insects as food [38], was not the main feeling associated with the use of insects as feed. Moreover, the sense of disgust was significantly reduced by providing consumers with more information. Moreover, when compared to current conventional feed sources (e.g., fish meal and GM soymeal), the insect meal was strongly preferred as a potential feed for farmed ducks. 

Italian consumers are becoming increasingly interested in research on more sustainable feeds [17]. Our results show that these consumers are open to accepting changes to current animal production systems—for instance, replacing conventional protein feed (e.g., GM soy) with alternative, novel feedstuff. 

However, the general public is still unaware of the potential benefits of this alternative protein source for farmed animals, and the role of information may be greater for individuals who are uninformed or misinformed about the benefits of insect-based feed. Legislators should consider this lack of knowledge and help inform consumers about the benefits of insect meals, such as reducing the environmental impact of traditional feed (e.g., land use changes and deforestation), providing a natural and highly nutritious food-source, and helping reduce the burden of increasing demands for meat. Policy-makers should also consider whether mandatory labeling of such products is desirable and how this labeling could play a role in consumers’ choices [23]. For instance, an insect-fed certification could convey high animal welfare standards [25] and appeal to consumers concerned about the types of feed given to animals. Notably, live insects may gain wider acceptance than insect meal due to the former being perceived as a more natural type of feed for poultry than the latter. As live insects are currently allowed to be used as feed for several farmed animals (while processed insects remain more restricted), the positive consumer response to the use of live insect larvae could be associated with organic poultry production. The use of live insects would also improve the wellbeing of the animals.

However, the present study is not without limitations. First, the sample size was relatively small and not representative of the Italian population (e.g., the percentage of consumers with a tertiary level of education was higher than the national average). Therefore, generalizing the results to the entire Italian population would be difficult. Secondly, not many respondents were familiar with duck consumption. Nevertheless, focusing on a specific product, duck meat, provided a more concrete context for the respondents, even for a hypothetical situation. Moreover, to the best of our knowledge, this is the first study exploring Italian consumers’ opinions towards a poultry animal fed with insects, thereby providing new evidence on how potential consumer responses differ between the use of insect-based feed and live insects as feed. 

Although a study by Cullere et al. [42] showed comparable meat quality and sensory traits between poultry fed with Black Soldier Fly (*Hermetia illucens* L.) larvae and poultry fed soybean oil, future studies should integrate a multidisciplinary approach including both consumer and sensory sciences to explore the role of information in influencing sensory attributes and perception. Thus, future research should investigate consumers’ perceptions along with their sensory experiences [13,24], including blind, expected, and informed test conditions, which could help marketers better label insect-fed animal products.

## 5. Conclusions

This study shows that information about the benefits of insects as feed could improve consumers’ attitudes towards animal-based products fed with insects. 

In the Global North, entomophagy has received global media attention in recent years, contributing to an increase in curiosity among consumers and providing publicity for the private sector [43]. The same phenomenon might happen for this novel feedstuff in the near future.

Our findings provide clues for how the feed industry could adapt its communication to target audiences. As suggested by Ankamah et al. [18], the provision of information through public campaigns and marketing on the sustainability of insect feed could increase positive attitudes towards this new alternative feed source and create positive preferences. Beyond sustainability, our results suggest that the use of insects as feed will likely be accepted if consumers are reassured that the final product will be effectively safe and healthy and that the price and taste will remain unchanged. 

## Figures and Tables

**Table 1:** Willingness to purchase duck fed with different feed sources ^1^.

Different Types of Feed	Median (IQR)	Mean	SD
Duck fed with cereals	7.00 (2.00)	5.71	1.74
Duck fed with non-genetically modified (non-GM) soybean meal	6.00 (3.00)	5.08	1.97
Duck fed with insect meal	6.00 (3.00)	5.00	2.06
Duck fed with fish meal	4.00 (4.00)	3.81	2.08
Duck fed with genetically modified (GM) soybean meal	4.00 (5.00)	3.76	2.32

^1^ Measured on a 7-point scale from 1 (extremely unlikely) to 7 (extremely likely). IQR: interquartile range; SD: standard deviation.

**Table 2:** Attitude towards and intention to purchase a farmed duck fed via different feeding methods (median, IQR: interquartile range; mean, SD: standard deviation; *p*-value for non-parametric Mann–Whitney test for independent samples).

Treatment	Item	Control (*n* = 279)			Treatment (*n* = 286)			*p*-Value
		Median (IQR)	Mean	SD	Median (IQR)	Mean	SD	
Pre	Attitude towards eating a farmed duck fed with insect-based meal	4.60 (1.20)	4.45	1.22	4.60 (1.20)	4.54	1.18	0.240
Post	Attitude towards eating a farmed duck fed with insect-based meal	5.00 (1.67)	4.82	1.28	5.33 (1.50)	5.16	1.24	<0.001
Post	Attitude towards eating a farmed duck fed with live insects	4.83 (2.00)	4.71	1.53	5.33 (1.50)	5.10	1.37	0.001
Pre	Intention to purchase a farmed duck fed on an insect-based meal	6.00 (3.00)	4.98	2.05	6.00 (3.00)	5.02	2.07	0.741
Post	Intention to purchase a farmed duck fed on an insect-based meal	6.00 (3.00)	5.27	1.68	6.00 (3.00)	5.57	1.60	0.014
Post	Intention to purchase a farmed duck fed with live insects	6.00 (3.00)	5.15	1.91	6.00 (2.00)	5.58	1.69	0.008
Post	Intention to purchase a farmed duck fed on a vegetable meal diet	6.00 (3.00)	5.52	1.58	6.00 (3.00)	5.35	1.74	0.404
Post	Intention to purchase a wild duck	6.00 (3.00)	5.35	1.95	6.00 (3.00)	5.19	1.98	0.313

**Table 3:** Attitude towards, intention to purchase, and willingness to pay (WTP) for a farmed duck fed with insects between males and females (median, IQR: interquartile range; mean, SD: standard deviation; *p*-value for non-parametric Mann–Whitney test for independent samples).

Item	Males (*n* = 258)			Females (*n* = 300)			*p*-Value
	Median (IQR)	Mean	SD	Median (IQR)	Mean	SD	
Attitude towards eating a farmed duck fed with insect-based meal	5.17 (1.50)	5.15	1.08	5.00 (1.83)	4.88	1.41	0.074
Attitude towards eating a farmed duck fed with live insects	5.17 (1.67)	5.11	1.29	5.00 (2.00)	4.73	1.59	0.011
Intention to purchase a farmed duck fed with insect-based meal	6.00 (2.00)	5.66	1.47	6.00 (3.00)	5.23	1.77	0.008
Intention to purchase a farmed duck fed with live insects	6.00 (2.00)	5.70	1.56	6.00 (3.00)	5.08	1.97	<0.001
WTP for a farmed duck fed with insect-based meal	8.95 (0.00)	8.90	1.80	8.95 (0.00)	8.84	1.77	0.850
WTP for a farmed duck fed with live insects	8.95 (0.00)	8.92	1.86	8.95 (0.00)	8.93	1.94	0.953

**Table 4:** Attitude towards, intention to purchase, and willingness to pay (WTP) for a farmed duck fed with insects, by educational level (median, IQR: interquartile range; mean, SD: standard deviation; *p*-value for non-parametric Mann–Whitney test for independent samples).

Item	Secondary ^1^ (*n* = 106)			Tertiary ^2^ (*n* = 453)			*p*-Value
	Median (IQR)	Mean	SD	Median (IQR)	Mean	SD	
Attitude towards eating a farmed duck fed with insect-based meal	5.00 (2.00)	4.75	1.41	5.17 (1.50)	5.06	1.23	0.047
Attitude towards eating a farmed duck fed with live insects	5.00 (2.04)	4.72	1.70	5.00 (2.00)	4.96	1.40	0.428
Intention to purchase a farmed duck fed on an insect-based meal	5.00 (2.00)	4.96	1.72	6.00 (2.00)	5.54	1.61	<0.001
Intention to purchase a farmed duck fed with live insects	5.00 (3.00)	4.89	2.02	6.00 (2.00)	5.48	1.74	0.005
WTP for a farmed duck fed on an insect-based meal	8.95 (0.00)	8.93	1.94	8.95 (0.00)	8.86	1.71	0.922
WTP for a farmed duck fed with live insects	8.95 (0.22)	8.71	2.17	8.95 (0.00)	8.98	1.84	0.127

^1^ Secondary education included a high school diploma. ^2^ Tertiary education included university and postgraduate education.

## Data Availability

The data presented in this study are available upon reasonable request from the corresponding author.

## Appendix A

**Table A1:** Items for measuring consumers’ attitudes towards eating a farmed duck fed with insect-based meal—pre-information treatment.

I Believe That Using Insects as Feed for Ducks: ^1^	Control (*n* = 279)			Treatment (*n* = 286)		
	Median (IQR)	Mean	SD	Median (IQR)	Mean	SD
… will negatively–positively affect the taste of the meat	4.00 (1.00)	4.15	1.31	4.00 (1.00)	4.16	1.37
… will negatively–positively affect the nutritional properties of the meat	4.00 (2.00)	4.47	1.56	4.00 (1.00)	4.53	1.50
… negatively–positively affect the taste of the final duck-based products (e.g., duck sausages)	4.00 (0.00)	4.08	1.33	4.00 (1.00)	4.09	1.33
… is extremely disgusting–not at all disgusting	4.00 (1.00)	4.04	1.59	4.00 (1.00)	4.14	1.49

^1^ All items were measured on a 7-point Likert scale.

**Table A2:** Examples of respondent comments based on the different responses and treatments.

‘Would You Eat Farmed Duck Fed on an Insect-Based Diet?’	
Control Group (*n* = 279)	Treatment Group (*n* = 286)
Yes, because… (*n* = 189)	Yes, because… (*n* = 225)
*“it can improve animal welfare, natural feed, maybe more sustainable”, “I think duck is eating insects in its natural environment”, “Yes, because I don’* *t think it influences the meat quality”, “I think insects are part of the ordinary animal diet”, “Yes, because I think it’* *s an eco-friendly choice in the environmental impact”, “Taste could be better”, “Maybe it’* *s more eco-friendly”, …*	*“I think it is a natural diet”, “ducks already eat insects”, “it makes sense”, “it is what they would naturally eat”, “I do not see any problems/drawbacks”, “in nature a wild duck also eats insects and other animal organisms”, “low environmental impact”, “it is more sustainable”, “from what I read it is seems a positive thing”, “I believe it has environmental and economic benefits”, “the taste of the meat do not change”, “better animal welfare”, “it is safe and ethical”, “I am curious about tasting it”, …*
Yes, but … (*n* = 37)	Yes, but … (*n* = 32)
*“it should be reported the safety of insects”, “I’* *d like to read a declaration about flour safety”, “info shown on the label”, “with labels that certified the animal nutrition”, “the taste and nutrition aspects should be similar”* *, “I’* *m not sure about organoleptic and nutritional qualities”, “Yes, only if this kind of nutrition doesn’* *t have an effect on sensorial characteristics of the ended product”, “Taste must be identical or better than “traditional duck”, …*	*“if studies confirm the safety of insects”, “if it is healthy”, “I would need more information on how insects are farmed”, “if the info would be shown on the label”, “only if the insects are part of the life cycle of ducks”, “I would like to have a certification of origin and safety of insects”, “it depends from the insect species”, …*
Maybe, if… (*n* = 24)	Maybe, if… (*n* = 11)
*“the taste is not altered”, “I would be more informed about this feed”, “quality and safety of meat don* *’* *t change”, “I* *’* *m sure it doesn* *’* *t represent a problem in meat safety”, “If I knew more about it...”, “if it was not too expensive and if it was better than other animals from an ecological point of view”, …*	*“some other people will try first* *”, “there was not threat to my health”, “I were informed about the pros and cons”, “the duck already eats insects in nature”, “the taste and the nutrition quality of the meat would not be altered”, “I have to be sure that there will not be allergic reactions”, …*
No, because… (*n* = 29)	No, because… (*n* = 18)
*“I am not sure whether Duck is eating insects”, “I do not like the idea”, “it makes me disgust”, “I* *’* *m a little disgusted”, “I would be disgusted If I knew I* *’* *m eating insects”, “I do not consume duck”, “I don* *’* *t like duck”, “It* *’* *s cultural”, “I don* *’* *t eat duck more if it is feeding with insects”, “I* *’* *m not sure if ducks are insectivorous”, …*	*“only thinking about that it makes me disgust”, “no absolutely”, “I do not consume duck”, “I don* *’* *t like duck meat”, “I feel disgust”, “a new production method implies some risks”, …*
